# Chemerin levels in chronic kidney disease: A systematic review and meta-analysis

**DOI:** 10.3389/fendo.2023.1120774

**Published:** 2023-01-25

**Authors:** Amir Hossein Behnoush, Parnian Shobeiri, Pegah Bahiraie, Nikan Amirkhani, Amirmohammad Khalaji, Soheil Peiman

**Affiliations:** ^1^ School of Medicine, Tehran University of Medical Sciences, Tehran, Iran; ^2^ Non–Communicable Diseases Research Center, Endocrinology and Metabolism Population Sciences Institute, Tehran University of Medical Sciences, Tehran, Iran; ^3^ Network of Immunity in Infection, Malignancy and Autoimmunity (NIIMA), Universal Scientific Education and Research Network (USERN), Tehran, Iran; ^4^ Research Center for Immunodeficiencies, Pediatrics Center of Excellence, Children’s Medical Center, Tehran University of Medical Sciences, Tehran, Iran; ^5^ School of Medicine, Shahid Beheshti University of Medical Sciences, Tehran, Iran; ^6^ Department of Internal Medicine, AdventHealth Orlando Hospital, Orlando, FL, United States

**Keywords:** chemerin, chronic kidney disease, renal disease, systematic review, meta-analysis

## Abstract

**Introduction:**

Chemerin as an inflammatory biomarker has gained attention in its biomarker capability. Several studies measured its levels in chronic kidney disease (CKD), as one of the common non-communicable causes of mortality and morbidity. Hence, this systematic review and meta-analysis aimed to investigate this association.

**Methods:**

PubMed, Scopus, Embase, and the Web of Science databases were systematically searched for studies investigating chemerin levels in any CKD stage (including end-stage renal disease patients undergoing hemodialysis (HD)) and comparing it with healthy controls. Random effect meta-analysis was performed to calculate the standardized mean difference (SMD) and 95% confidence interval (CI).

**Results:**

A total of eight studies were included, comprised of 875 individuals, with a mean age of 56.92 ± 11.78 years. All studies had high quality based on the New Castle-Ottawa Scale (NOS). Meta-analysis revealed significantly higher levels of chemerin in CKD patients compared to healthy controls (SMD 2.15, 95% CI 0.83-3.48, p-value<0.01). Additionally, HD patients had statistically higher levels of chemerin than controls (SMD 2.10, 95% CI 0.58-3.62, p-value=0.01). In meta-regression, publication year accounted for 23.50% and 24.17% of heterogeneity for these analyses, respectively.

**Conclusion:**

Chemerin can be potentially used as a biomarker in CKD patients, which can suggest the inflammatory pathways for the disease. Further research is warranted for the assessment of its clinical applications and enlightening its role in the pathophysiology of CKD.

## 1 Introduction

Chronic kidney disease (CKD) is a significant contributor to noncommunicable disease morbidity and mortality. More than 10% of the adult population had markers for renal disease, according to large-scale, nationally representative screening programs carried out in the 2000s (1). This chronic disease is characterized by a permanent serious impairment of kidney function and a reduced glomerular filtration rate (GFR) for at least three months, which results in a loss of the kidneys’ normal ability to remove toxins from the body. Renal injury markers, such as urinary and hematological changes, can be used to detect this impairment (2). Biomarkers can be used as tools for screening, diagnosing, and monitoring diseases as well as evaluating the response to therapeutic interventions (3).

Chemerin was initially identified as a chemokine found in the inflammatory fluids of cancer and rheumatoid arthritis patients (4). It is also known by the names tazarotene-induced gene 2 (TIG2) and retinoic acid responder 2 (RARRES2). Chemerin and its receptor CMKLR1 appear to control insulin sensitivity, adipocyte differentiation, and glucose and lipid balance (5, 6). Adipose tissue, liver, platelets, placenta, and to a lesser degree, other tissues such as the kidneys have all been discovered to express it. The connection between chemerin and renal function has also drawn more attention recently (7). This marker is thought to influence the beginning and development of the local inflammatory state. The inflammatory cells that have been triggered release the enzymes that convert the circulating pro-chemerin to chemerin. Other immune cells are drawn to the area of inflammation by this, which strengthens their adherence (8).

Some studies reported that kidney function is inversely related to circulating chemerin in CKD patients. Blaszak et al. expressed that the mean serum chemerin level in stages 3 and 4 of CKD was 70% higher than the control group and according to a study conducted by Rutkowski et al., the serum chemerin concentration decreased to values observed in control subjects after successful kidney transplantation (6, 8). In another study, Sarhat et al. found that the concentration of chemerin was significantly lower in patients with renal failure, compared to controls (2).

To combine recent data of investigations on chemerin in recent years, addressing the link of this unique marker with CKD, a thorough evaluation, and meta-analysis of original research publications were undertaken in this study.

## 2 Methods

### 2.1 Search strategy

In November 2022, a comprehensive search of worldwide web databases, including PubMed, SCOPUS, Web of Science, and Embase, was conducted. The keywords utilized were “chemerin” AND “chronic renal disease”, in addition to other pertinent keywords, which were explained in detail in **Supplementary Table 1**. The search was performed without any restrictions or filters.

### 2.2 Inclusion and exclusion criteria

The inclusion criteria were: 1) studies assessing chemerin levels in patients with CKD and controls and 2) studies evaluating chemerin levels in patients with CKD undergoing dialysis (hemodialysis (HD)) and controls. We excluded: 1) studies without a control group; 2) studies without exact concentration of chemerin levels, after emailing the corresponding author; 3) non-English articles; and 4) letters, commentaries, case reports, conference abstracts, and reviews.

### 2.3 Screening

Two reviewers (AHB and PB) individually reviewed titles and abstracts for relevant articles based on inclusion and exclusion criteria, after eliminating duplicates from the initial search. Then, the entire texts of included papers were evaluated, and in cases of disagreement, a discussion with the third reviewer (AK) finalized the conclusion. Lastly, the references to the included papers were investigated.

### 2.4 Data extraction

Using a data extraction sheet, two independent reviewers (AHB and PS) extracted the following information from each study: 1) First author’s name, publication year, and country of conduct; 2) the demographic characteristics of the cases (sample size, mean age, and gender distribution in each CKD and control group); 3) plasma and/or serum chemerin concentration in each group; 4) chemerin gene polymorphism alleles for each study. In situations where precise data regarding the concentration of chemerin were unavailable, we contacted the corresponding author of the investigations.

### 2.5 Quality assessment

The “Newcastle-Ottawa Quality Assessment Scale” (NOS) for observational studies was used for the quality assessment of included studies (9). Two authors did the quality evaluation individually. In the event of a dispute, a third author settled the issue. The NOS contains three primary classifications of bias: selection, comparability, and outcome. Studies with quality scores of 9-10, 7-8, 5-6, and less than 5 were deemed “very good,” “good,” “satisfactory,” and “unsatisfactory,” respectively.

### 2.6 Statistical analysis

Random-effect meta-analysis was used to determine the standardized mean difference (SMD) and 95% confidence interval (CI) of chemerin concentrations in CKD and control groups. The p-values <0.05 were used as the cutoff for statistical significance.

Where median and interquartile range or median and range were presented in the studies, they were transformed to mean and standard deviations (SDs) following the methods recommended by Luo et al. and Wan et al. (10, 11). In addition, means and standard deviations were combined where necessary, as suggested by the Cochrane handbook (12).

To calculate the heterogeneity, we utilized Higgins’ I-square test based on Cochrane’s Q. The heterogeneity thresholds for low, moderate, and high heterogeneity were 25%, 26-75%, and 75%, respectively. Due to the high heterogeneity among studies, random-effect meta-analysis (restricted maximum likelihood (REML)) was employed. We performed a sensitivity analysis by omitting each study and examining the effect on the total effect size. To identify potential outliers, Galbraith plots were also used and analyzed. To identify the source of heterogeneity, meta-regression of the sample size, mean age, female percentage, and publication year was also performed. In addition to Egger’s and Begg’s statistical tests, publication bias was evaluated using a visual examination of funnel plots (13, 14).

## 3 Results

### 3.1 Literature search and baseline characteristics of included studies

**Figure 1:**
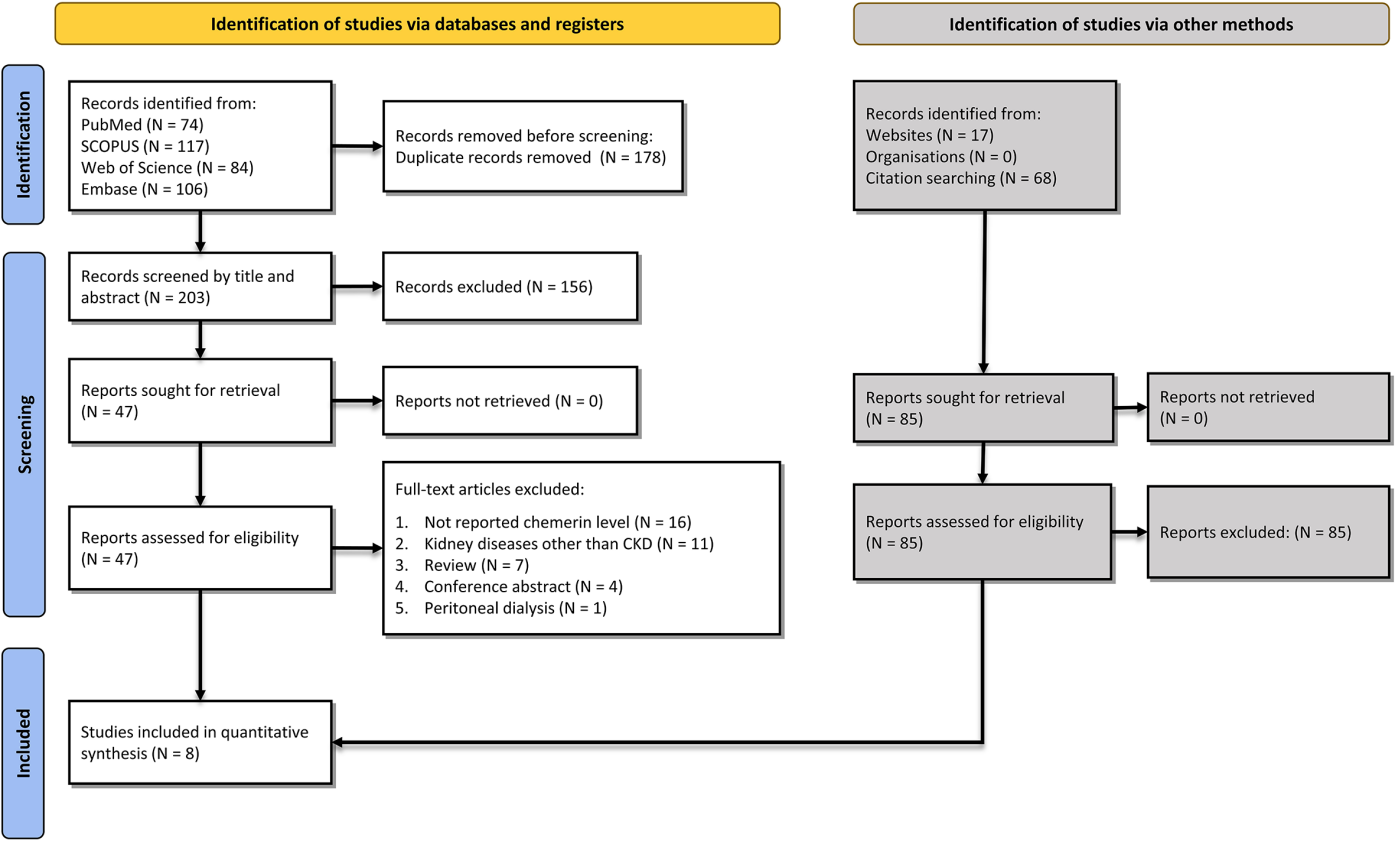
Flow diagram summarizing the selection of eligible studies based on the PRISMA guidelines.

Our search included 381 records from PubMed (n = 74), SCOPUS (n = 117), Web of Science (n = 84), and Embase (n = 106). After removing 178 duplicates, 156 records were excluded based on title and abstract screening. Assessment of full-texts resulted in excluding 39 articles due to not reporting chemerin levels, not assessing chemerin levels in CKD, review articles, and conference abstracts. Details of the identification of studies are illustrated in **Figure 1**.

Eight studies with 875 participants were included in our study that measured serum levels of chemerin in CKD patients and controls (2, 6, 8, 15–19) (**Table 1**). The mean age of participants was 56.92 ± 11.78 years and 58.6% were male. Studies were conducted in Poland (6, 8), Egypt (15, 19), Iraq (2, 16), China (17), and Germany (18) between 2009 and 2022. All studies had good quality based on the NOS system, while two of them had very good quality due to high comparability among studies groups (15, 19) (**Supplementary Table 2**).

**Table 1:** Characteristics of studies evaluating the relation between chemerin levels and chronic kidney disease.

Author	Year	Location	Specimen	Population	N Total	Age (years)	Male (%)	Findings
***Blaszak et al.***	2015	Poland	Serum	CKD stages 3&4), HD, KT, Control	187	67.5 ± 12.1	60.96	The mean serum chemerin level in the CKD group (stages 3&4) was 70% higher than in controls (122.9 ± 33.7 vs. 72.6 ± 20.7 ng/mL; p<0.001). In addition, no statistical difference was observed between HD patients and CKD ones (115.7 ± 17.6 vs. 122.9 ± 33.7 ng/mL; n.s.). The mean Chemerin levels significantly decreased after HD (115.7 ± 17.6 vs. 101.5 ± 16.4 ng/mL; p<0.001). Chemerin levels after HD were significantly higher than those with KT (101.5 ± 16.4 vs. 74.8 ± 16.0 ng/mL; p<0.001). There was also no significant difference between KT patients and controls (74.8 ± 16.0 vs. 72.6 ± 20.7 ng/mL; n.s.).
***El-Khashab et al.***	2019	Egypt	Serum	CKD stages 3&4, ESRD (HD), Control	80	49.5 ± 13.9	67.5	The mean chemerin level was significantly higher in CKD patients compared to the healthy controls (p<0.001).
***Fahad et al.***	2020	Iraq	Serum	HD with and without DM, T2DM, Control	120	54.0 ± 10.1	45.83	The mean serum chemerin was significantly higher in HD patients with DM (230.13 ± 78.26 ng/ml), followed by HD patients without DM (221.90 ± 65.17 ng/ml) compared with controls (110 ± 20.42 ng/ml). Also, the mean of serum chemerin significantly increased in DM patients (212.29 ± 70.88 ng/ml) when compared with the control.
***Liu et al.***	2022	China	Serum	CRF (GFR ≤ 60), Control	148	59.2 ± 8.1	66.89	Compared with the healthy group, the expression level of chemerin in the observation group was decreased, and the difference was statistically significant (p<0.001). Also, serum levels of chemerin in the death group were significantly higher than those in the survival group(p<0.001, 145.41 ± 18.75 vs 98.52 ± 14.92). The ROC-AUC analysis for the prediction of mortality resulted in an AUC of 0.775 (95% CI: [0.614-0.872]).
***Pfau et al.***	2009	Germany	Serum	CKD (GFR<50), Control	120	65.0 ± 17.5	51.67	Median circulating chemerin was more than two-fold higher in CKD patients (542.2 ± 98.1 μg/l) compared with control patients (254.3 ± 88.7 μg/l) (p<0.001). There was no significant difference between males and females or patients with or without T2DM.
***Rutkowski et al.***	2011	Poland	Serum	ESRD, Control	46	47.3 ± 13.6	60.86	Patients before KT had significantly higher chemerin levels compared to healthy controls. After KT, the chemerin levels were reduced to the range of healthy controls. In 76% of the patients, a decrease in serum chemerin concentration was observed; however, the percentage of the changes differed from 13% to 66% (p<0.05).
***Salama et al.***	2016	Egypt	Serum	Pre-HD, HD, Control	78	48.6 ± 8.6	NR	Compared with the control participants, pre-dialysis patients and patients on HD had significantly higher chemerin levels (p=0.001 and p<0.001). In addition, the chemerin level in patients on HD was higher than that in pre-dialysis patients (p<0.001).
***Sarhat et al.***	2018	Iraq	Serum	CRF (pre- and post-HD), Control	96	43.9 ± 7.6	57.29	CRF patients had significantly lower chemerin levels compared to controls. In addition, post-HD patients had increased levels of chemerin.

Data are presented as mean± standard deviation or percentage. NR, not reported; CI, confidence interval; HD, hemodialysis; CKD, chronic kidney disease; KT, kidney transplantation; ESRD, end-stage renal disease; DM, diabetes mellitus; T2DM, type-2 diabetes mellitus; GFR, glomerular filtration rate; CRF, chronic renal failure; AUC, area under the receiver operating characteristic curve.

### 3.2 Meta-analysis of chemerin levels in all-stage-CKD patients and controls

**Figure 2:**
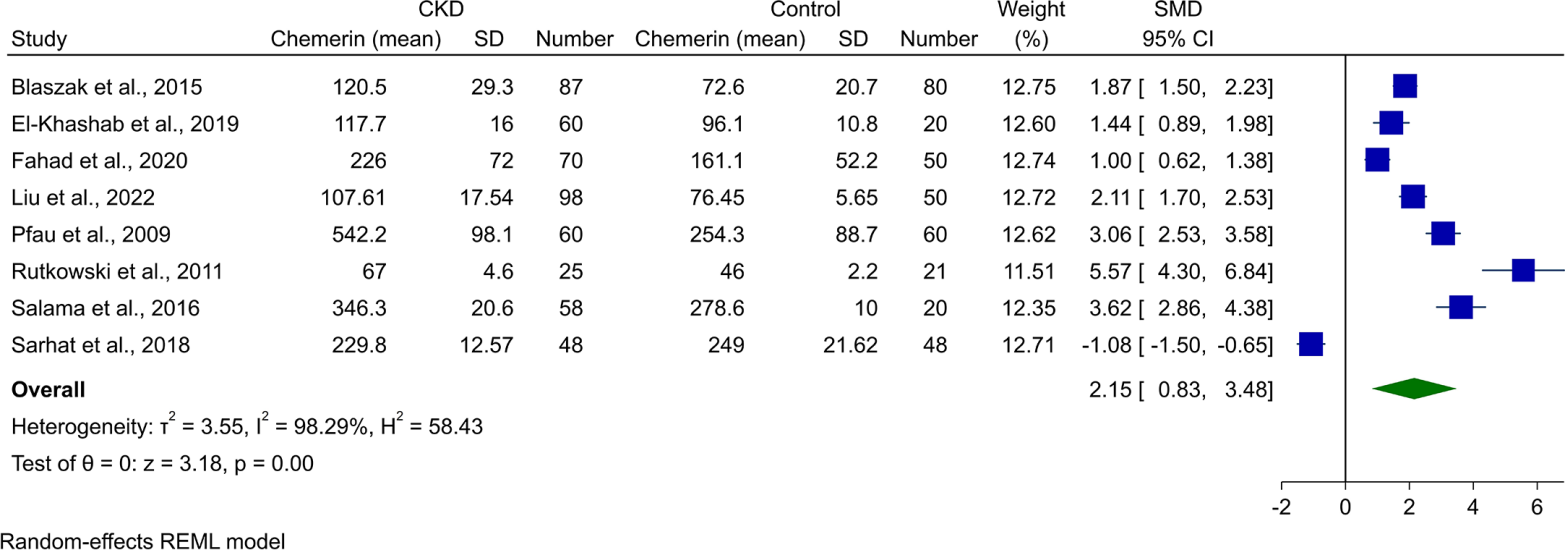
Forest plot for meta-analysis of chemerin levels in chronic kidney disease patients compared to controls.

Pooling of the eight studies comparing CKD in any stage and healthy controls revealed significantly higher chemerin levels in CKD patients (SMD [95% CI]: 2.15 [0.83, 3.48], p-value <0.01, **Figure 2**). However, this was associated with high heterogeneity (*I* ^2^: 98.29%).

### 3.3 Meta-analysis of chemerin levels in HD patients and controls

**Figure 3:**
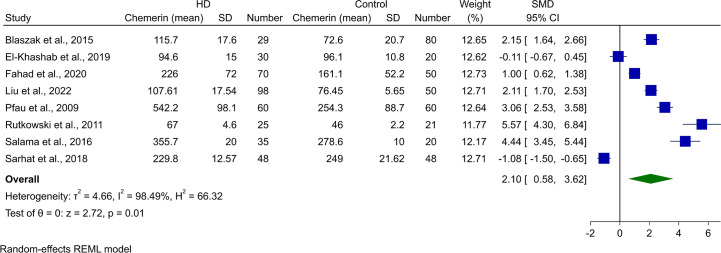
Forest plot for meta-analysis of chemerin levels in hemodialysis patients compared to controls.

Assessment of chemerin levels in HD patients in comparison with controls was done in eight of the studies. Random-effect meta-analysis showed statistically higher chemerin blood concentrations in HD cases (SMD [95% CI]: 2.10 [0.58, 3.62], p-value: 0.01, **Figure 3**). Heterogeneity was also high in this analysis (*I* ^2^: 98.49%).

### 3.4 Meta-analysis of chemerin levels in non-HD CKD patients and controls

**Figure 4:**
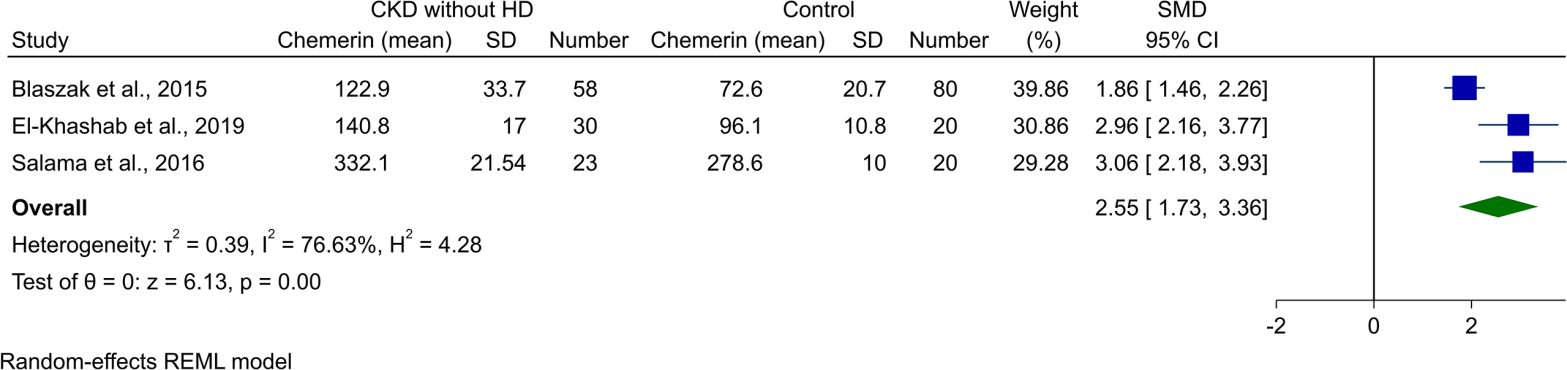
Forest plot for meta-analysis of chemerin levels in chronic kidney disease patients without hemodialysis compared to controls.

Three of the studies reported chemerin levels in CKD patients who were not undergoing HD and compared it with healthy controls. Blaszak et al. (8) and El-Khashab et al. (15) investigated stage 3 and 4 CKD patients, while Salama et al. (19) reported levels in CKD patients on conservative treatment. Meta-analysis of comparison with healthy controls resulted in significantly higher levels of chemerin (SMD [95% CI]: 2.55 [1.73, 3.36], p-value<0.01, *I* ^2^: 76.63%, **Figure 4**).

### 3.5 Publication bias

Publication bias was assessed for CKD vs. control and HD vs. control meta-analyses. While there was no apparent symmetry for the former, the latter showed an asymmetry in the funnel plot (**Supplementary Figures 1**, **2**). Begg’s statistical test could not reveal any sign of publication bias in either CKD vs. controls (p-value: 0.173), or HD vs. controls (p-value: 0.265); however, Egger’s test showed a significant publication bias for both of them (p-value: 0.002 and 0.001, respectively).

### 3.6 Meta-regression analysis

Meta-regression was performed on possible modifiers, including sample size, mean age, male percentage, and publication year. Univariable meta-regression showed no significant relationship between any of these and two of the main analyses. Additionally, publication year contributed to 44.19% and 24.17% of the observed heterogeneity in CKD vs. control and HD vs. control meta-analyses, respectively (**Table 2**). Bubble plots for all mentioned meta-regressions are illustrated in **Supplementary Figures 3**-**10**.

**Table 2:** Meta-regression of CKD/Controls and HD/Controls meta-analyses.

Moderator	No. of subjects		Meta-regression				R^2^ Analog (proportion of variance explained)
	Case	Control	Slope	95% CI		*p-value*	
CKD vs. Control							
**Sample Size**	506	349	-0.018	-0.055	0.018	0.317	0%
**Age (mean, years)**	506	349	-0.024	-0.151	0.200	0.785	0%
**Male Percentage**	448	329	0.034	-0.183	0.251	0.758	0%
**Publication year**	506	349	-0.254	-0.534	0.026	0.075	23.50%
HD vs. Control							
**Sample Size**	395	349	-0.015	-0.060	0.029	0.495	0%
**Age (mean, years)**	395	349	0.036	-0.163	0.236	0.720	0%
**Male Percentage**	360	329	0.000	-0.237	0.236	0.997	0%
**Publication year**	395	349	-0.290	-0.608	0.027	0.073	24.17%

CI, confidence interval; CKD, chronic kidney disease; HD, hemodialysis.

### 3.7 Outlier’s detection and sensitivity analysis

Galbraith plots for both CKD/HD vs. control analyses were designed and investigated. No outlier study was identified in any of the two analyses (**Supplementary Figures 11**, **12**). In addition, sensitivity analysis was performed by removing each of the studies and assessing its impact on overall results. Similarly, none of the studies had a significant effect on the overall pooled result.

## 4 Discussion

CKD is a global health burden that is strongly associated with a decreased quality of life and premature mortality. It is usually defined using the serum creatinine concentration to estimate GFR as an index of kidney function (20). However, commonly used formulas to estimate kidney function based on creatinine alone have severe shortcomings, with an accuracy of less than 65%, according to estimates (21). These failures point to the need for additional markers of kidney function to augment current clinical tools. An example of a successful marker in diagnosing CKD is cystatin C, which is independent of patient muscle mass and has been used successfully alone and together with creatinine in new equations for estimating GFR that outperformed traditionally used equations (22, 23). We investigated whether chemerin could play a similar role.

The observed elevation in chemerin levels can reasonably be thought to be a result of either an increase in chemerin production or a decrease in its excretion. Chemerin is expressed throughout the body, but its production is believed to be dominated by the liver, with adrenal and pancreatic glands as additional significant sources (24). Comparatively, the expression of chemerin in the kidney is less than 5% of that in the liver (25). Alternatively, adipose tissue is thought to be the main source of chemerin in the body (26), which has led to the investigation of chemerin expression in subcutaneous adipose tissue in CKD patients in one of our included studies (8). The results showed no change in tissue expression, despite increased chemerin levels. While this result shows that subcutaneous chemerin expression is not the source of increased chemerin levels in CKD patients, it does not necessarily establish that an increase in chemerin expression throughout the body, e.g., by visceral adipose tissue, might not be the cause of the observed increase in chemerin. Additional studies are required to investigate chemerin expression in the main chemerin-producing tissues, including the liver, endocrine glands, and kidneys in patients with CKD.

The second mechanism by which the increased chemerin levels might be explained is the reduced excretion of chemerin due to impaired kidney function. This is the main mechanism by which creatinine and cystatin C are increased in kidney dysfunction as well (22). Chemerin, in its main form, is a 143 residue polypeptide weighing 16kDa, which lies within the range associated with decreased clearance in kidneys with impaired function (7). Elevated levels of adipokines, including leptin, adiponectin, tumor necrosis factor-α (TNF-α), interleukin-6, resistin, visfatin, and angiotensinogen, with similar molecular shapes and weights to chemerin, have been observed in kidney dysfunction (27). Decreased renal function may increase serum adipokine levels by reducing renal elimination or degradation. A possible source of evidence for this mechanism is the significant negative association between GFR and chemerin levels in patients with ESRD as well as the restoration of chemerin levels after successful kidney transplantation (6). Stronger evidence is required to validate this hypothesis, most obviously by measuring urine chemerin levels to calculate chemerin clearance in CKD patients. This approach was considered in (8) but was not reported owing to measurement problems.

In addition to the unknown precise mechanism of increased chemerin levels in CKD, its role in the pathophysiology of renal failure is unclear. We have discussed reports of meaningful associations between chemerin levels and the degree and risk of kidney dysfunction. However, it is unclear whether this observation is simply a byproduct of reduced kidney function due to other means or if chemerin is, in fact, an active pathological agent. Adipokines, in general, seem to have both protective and degenerative effects on kidney function, with all but adiponectin causing CKD progression by mediating endothelial dysfunction, inflammation, fibrosis, and oxidative stress (27). Chemerin specifically produces its function by binding one of three receptors: chemerin receptor 1 (chem1), chemerin receptor 2 (chem2), or chemokine receptor-like 2 (CCRL2). Chem1, the most common chemerin receptor, is a Gi/Go protein of the G protein family, which inhibits the production of cAMP and promotes the production of IP3, calcium influx, and activation of phospholipase C, PI3 kinase, and MAPK pathways (28). Chem1 is highly expressed in the kidneys, much more so than chemerin itself, suggesting high sensitivity to chemerin produced elsewhere (24). This receptor has been shown to mediate the proinflammatory function of chemerin owing to its expression on macrophages and dendritic cells. The proinflammatory function of chemerin has additionally been confirmed in arthritis and psoriasis, firmly establishing the proinflammatory activity of chemerin (29). Inflammation, along with the modulation of endothelial damage characteristic of adipokines, has been recently confirmed in the kidneys of patients and rats with diabetic nephropathy (DN), where the expression of chemerin, chem1, and inflammatory factors was significantly increased in DN, implicating chemerin as an active agent in the pathophysiology of glomerular endothelial cell inflammation (30). Additional investigations are needed to shed light on the role that the chemerin/chem1 axis plays in causing kidney injury.

The observed association between chemerin and kidney disease is of importance in two general axes (1): as a result of kidney damage, itself either caused by reduced renal clearance or increased production, and (2) as a cause of kidney damage, through a variety of proinflammatory, endothelial, and oxidative mechanisms. These two axes, in turn, implicate chemerin both as a marker for CKD diagnosis, as well as a possible target for the treatment of a variety of kidney diseases.

The strengths of this study include precise compliance with PRISMA protocols, including a comprehensive search of relevant databases and the independent screening of search results by two reviewers. The measured outcomes are mostly identical and directly comparable. The major limitation of this analysis was the relevant differences among included studies’ populations and the small sample size of them, resulting in a limitation in the generalizability of the findings. Secondly, few subgroup analyses were performed, which introduces a possible source of bias. Third, we calculated mean and SDs from median and IQRs with the methods suggested by Luo et al. and Wan et al. (10, 11), which despite being used before, may add some limitations to the analyses. Finally, the lack of additional direct prognostic indices limits the scope of our reasoning regarding the appropriateness of chemerin as a prognostic marker for CKD.

## 5 Conclusion

Evidence from recent studies suggests that the cytokine chemerin is consistently elevated in patients diagnosed with CKD and in patients undergoing HD compared to healthy controls. This evidence can be used to justify the inclusion of chemerin as one of several possible biomarkers for future models used to classify patients into CKD vs. normal as well as to predict the future course of their disease. Evidence from additional studies is needed to solidify and confirm this observed association as well as to specify pitfalls, including specific comorbidities or covariates, that cause the observed association to fail to replicate.

## Data availability statement

The original contributions presented in the study are included in the article/**Supplementary Material**. Further inquiries can be directed to the corresponding author.

## Author contributions

AHB: Writing - original draft/Conceptualization/Formal analysis/Visualization, PS, PB, and NA: Writing - original draft/Review and Editing, Data curation, AK: Supervision/Conceptualization/Writing - Review and Editing, SP: Writing - Review and Editing. All authors contributed to the article and approved the submitted version 
