# Supplementary material for: Chemerin levels in chronic kidney disease: A systematic review and meta-analysis
Source: Front Endocrinol (Lausanne). 2023 Jan 25;14:1120774. doi: 10.3389/fendo.2023.1120774 (PMC9907439; doi:10.3389/fendo.2023.1120774)
Supplement: Supplementary file 1 [file DataSheet_1.docx]

**Supplementary Materials**

**Chemerin Levels in Chronic Kidney Disease: A Systematic Review and Meta-Analysis**

**Supplementary Table 1.** Search strategy for each database

| **Query** | | **Results**  **(31 October 2022)** |
| --- | --- | --- |
| ***PubMed*** | | |
| #1 | ("chemerin*" OR "retinoic acid receptor responder protein 2" OR "tazarotene-induced gene 2 protein" OR "RAR-responsive protein" OR "RARRES2" OR "TIG2") | 1,363 |
| #2 | (("kidney"[tiab] OR "renal"[tiab] OR "hemodialysis"[tiab] OR "haemodialysis"[tiab] OR "dialysis"[tiab] OR “ESRF”[tiab] OR “ESKF”[tiab] OR “ESRD”[tiab] OR “ESKD”[tiab] OR “CKD"[tiab] OR “CKF”[tiab] OR “CRF”[tiab] OR “CRD”[tiab] OR “CAPD”[tiab]) OR ("Renal Insufficiency, Chronic"[Mesh] OR “Kidney”[Mesh] OR "Kidney Diseases"[Mesh] OR "Renal Insufficiency"[Mesh] OR "Renal Replacement Therapy"[Mesh] OR "Renal Dialysis"[Mesh] OR “Kidney Transplantation”[Mesh])) | 1,316,906 |
| **#3** | **#1 AND #2** | **74** |
| ***SCOPUS*** | | |
| #1 | (TITLE-ABS-KEY("chemerin*") OR TITLE-ABS-KEY("retinoic acid receptor responder protein 2") OR TITLE-ABS-KEY("tazarotene-induced gene 2 protein") OR TITLE-ABS-KEY("RAR-responsive protein") OR TITLE-ABS-KEY("RARRES2") OR TITLE-ABS-KEY("TIG2")) | 1,786 |
| #2 | (TITLE-ABS-KEY("kidney") OR TITLE-ABS-KEY("renal”) OR TITLE-ABS-KEY("hemodialysis”) OR TITLE-ABS-KEY("haemodialysis”) OR TITLE-ABS-KEY("dialysis”) OR TITLE-ABS-KEY(“ESRF”) OR TITLE-ABS-KEY(“ESKF”) OR TITLE-ABS-KEY(“ESRD”) OR TITLE-ABS-KEY(“ESKD”) OR TITLE-ABS-KEY(“CKD”) OR TITLE-ABS-KEY(“CKF”) OR TITLE-ABS-KEY(“CRF”) OR TITLE-ABS-KEY(“CRD”) OR TITLE-ABS-KEY(“CAPD”)) | 1,706,716 |
| **#3** | **#1 AND #2** | **117** |
| ***Embase*** | | |
| #1 | (("chemerin*”):ti,ab,kw OR ("retinoic acid receptor responder protein 2”):ti,ab,kw OR ("tazarotene-induced gene 2 protein”):ti,ab,kw OR ("RAR-responsive protein”):ti,ab,kw OR ("RARRES2”):ti,ab,kw OR ("TIG2”):ti,ab,kw) | 1,906 |
| #2 | (("kidney”):ti,ab,kw OR ("renal”):ti,ab,kw OR ("hemodialysis”):ti,ab,kw OR ("haemodialysis”):ti,ab,kw OR ("dialysis”):ti,ab,kw OR (“ESRF”):ti,ab,kw OR (“ESKF”):ti,ab,kw OR (“ESRD”):ti,ab,kw OR (“ESKD”):ti,ab,kw OR (“CKD”):ti,ab,kw OR (“CKF”):ti,ab,kw OR (“CRF”):ti,ab,kw OR (“CRD”):ti,ab,kw OR (“CAPD”):ti,ab,kw) | 1,498,532 |
| **#3** | **#1 AND #2** | **106** |
| ***Web of Science*** | | |
| #1 | (TS=("chemerin*") OR TS=("retinoic acid receptor responder protein 2") OR TS=("tazarotene-induced gene 2 protein") OR TS=("RAR-responsive protein") OR TS=("RARRES2") OR TS=("TIG2")) | 1,621 |
| #2 | (TS=("kidney") OR TS=("renal”) OR TS=("hemodialysis”) OR TS=("haemodialysis”) OR TS=("dialysis”) OR TS=(“ESRF”) OR TS=(“ESKF”) OR TS=(“ESRD”) OR TS=(“ESKD”) OR TS=(“CKD”) OR TS=(“CKF”) OR TS=(“CRF”) OR TS=(“CRD”) OR TS=(“CAPD”)) | 1,136,278 |
| **#3** | **#1 AND #2** | **84** |
| ***Total*** | | ***381*** |
| ***Total without duplicates*** | | ***203*** |

**Supplementary Table 2.** Quality Assessment of Included Studies Based on Newcastle-Ottawa Scale (NOS)

| **Study** | **Selection** | | | | **Comparability** | **Outcome** | | **Overall**  **Score** |
| --- | --- | --- | --- | --- | --- | --- | --- | --- |
|  | **Representation** | **Sample size** | **Non-Respondents** | **Exposure** |  | **Outcome** | **Statistical test** |  |
| **Blaszak et al.** | * | * | * | ** | - | ** | * | **8** |
| **El-Khashab et al.** | * | * | * | ** | ** | ** | * | **10** |
| **Fahad et al.** | * | * | * | ** | - | ** | * | **8** |
| **Liu et al.** | * | * | * | ** | - | ** | * | **8** |
| **Pfau et al.** | * | * | * | ** | - | ** | * | **8** |
| **Rutkowski et al.** | * | * | * | ** | - | ** | * | **8** |
| **Salama et al.** | * | * | * | ** | ** | ** | * | **10** |
| **Sarhat et al.** | * | * | * | ** | - | ** | * | **8** |


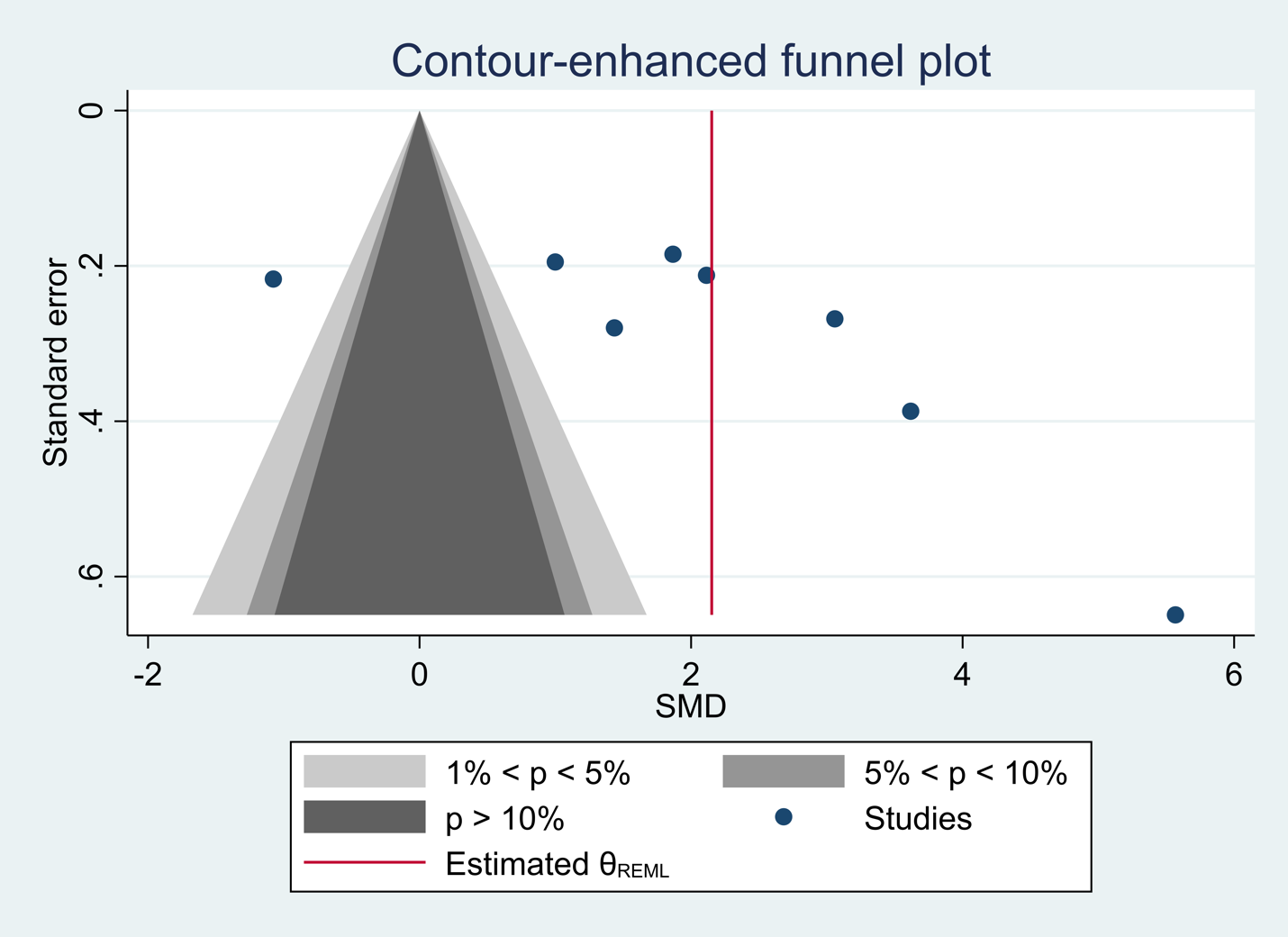


**Supplementary figure 1.** Funnel plot for analysis of chemerin levels in CKD patients vs. control


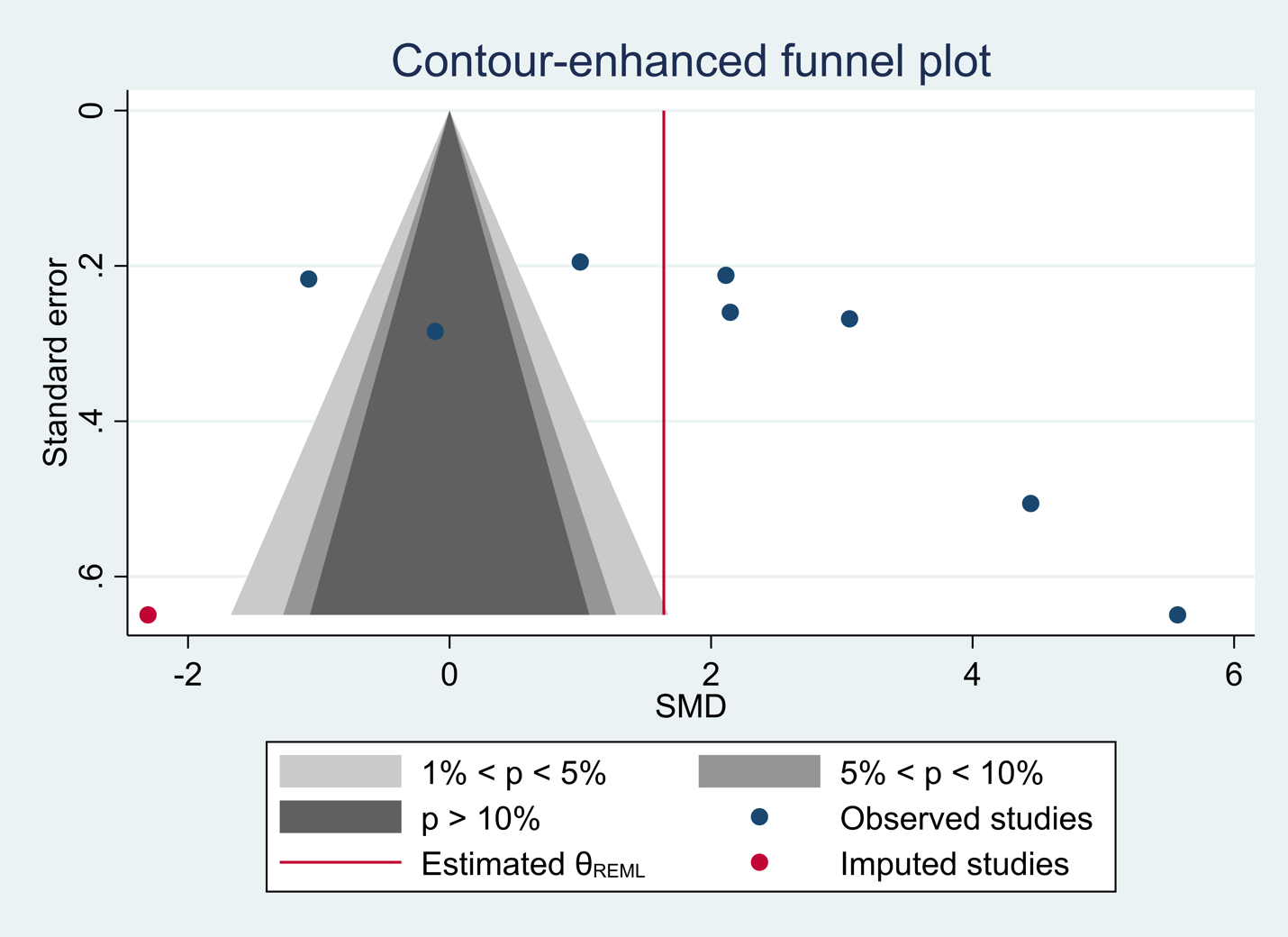


**Supplementary figure 2.** Funnel plot for analysis of chemerin levels in HD patients vs. control


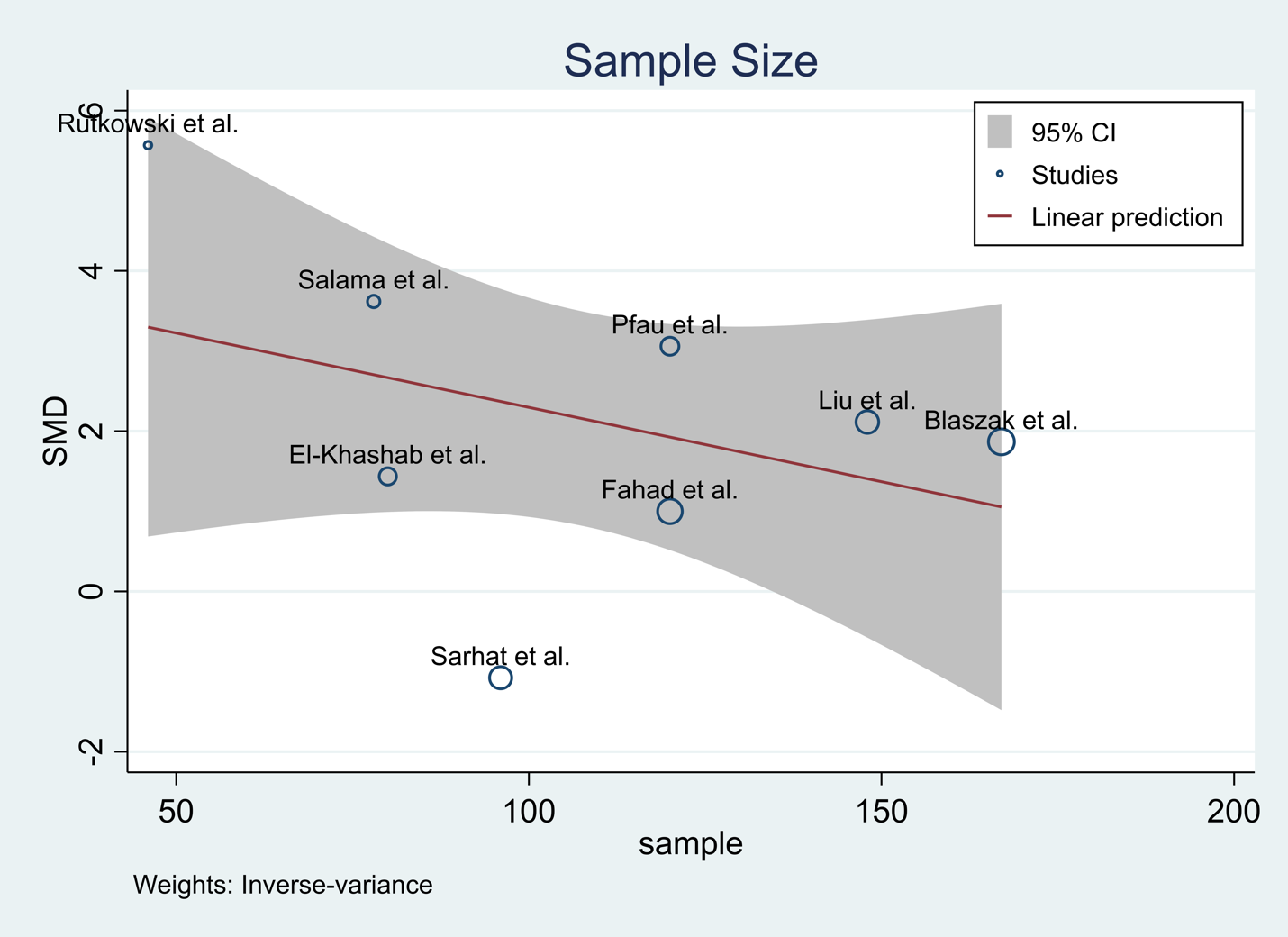


**Supplementary figure 3.** Bubble plot for meta-regression of CKD vs. controls based on sample size


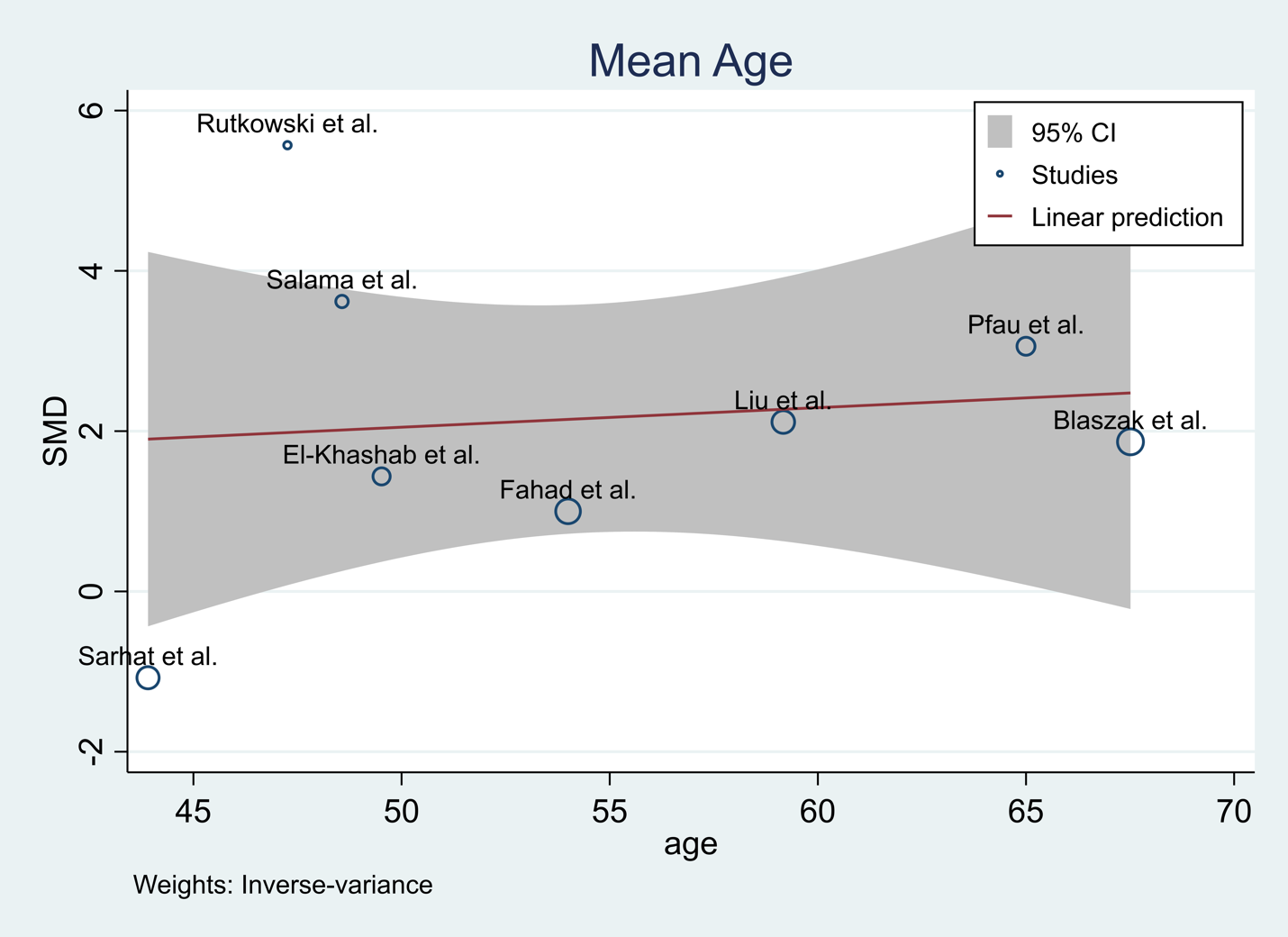


**Supplementary figure 4.** Bubble plot for meta-regression of CKD vs. controls based on mean age


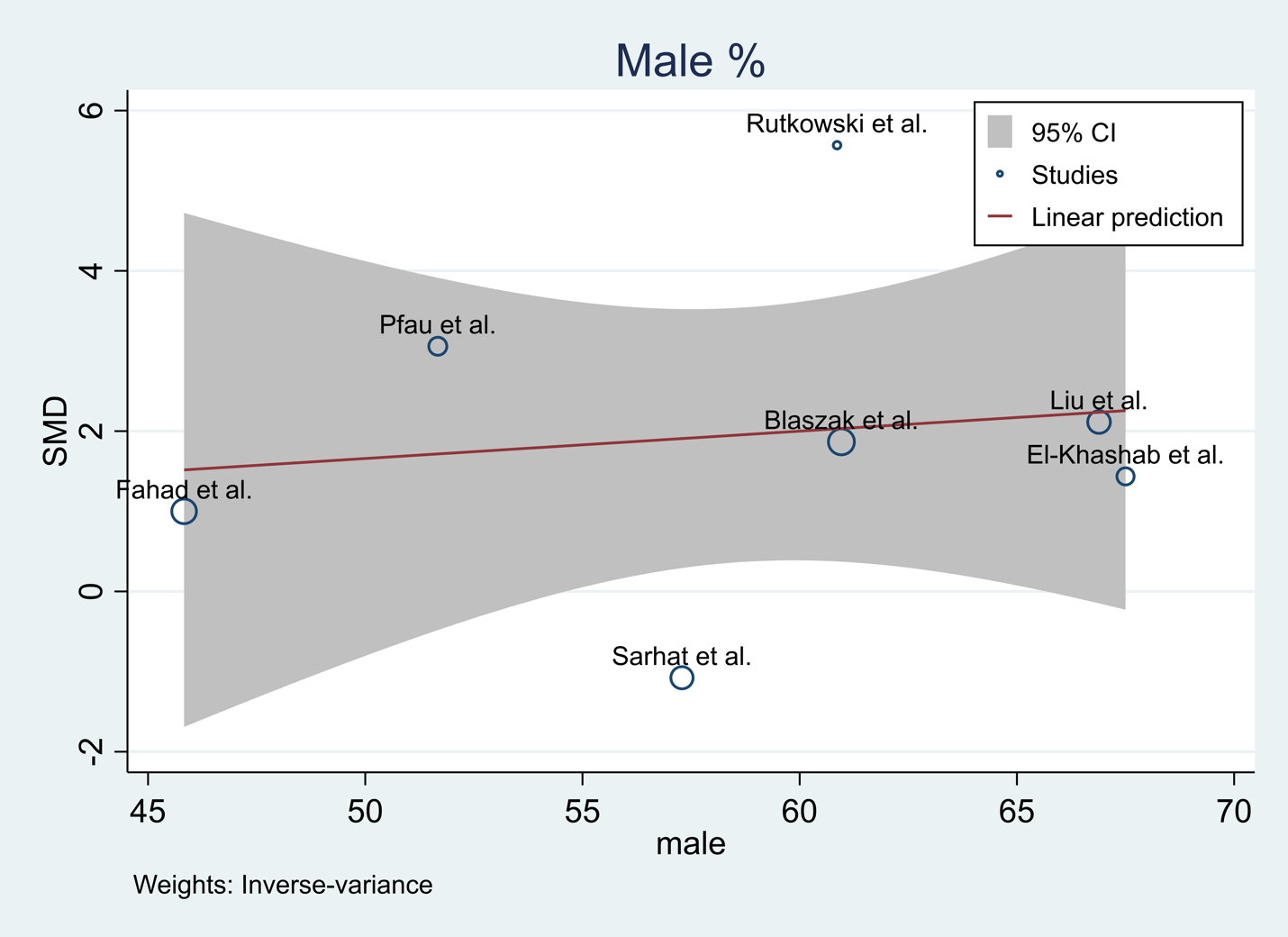


**Supplementary figure 5.** Bubble plot for meta-regression of CKD vs. controls based on male percentage


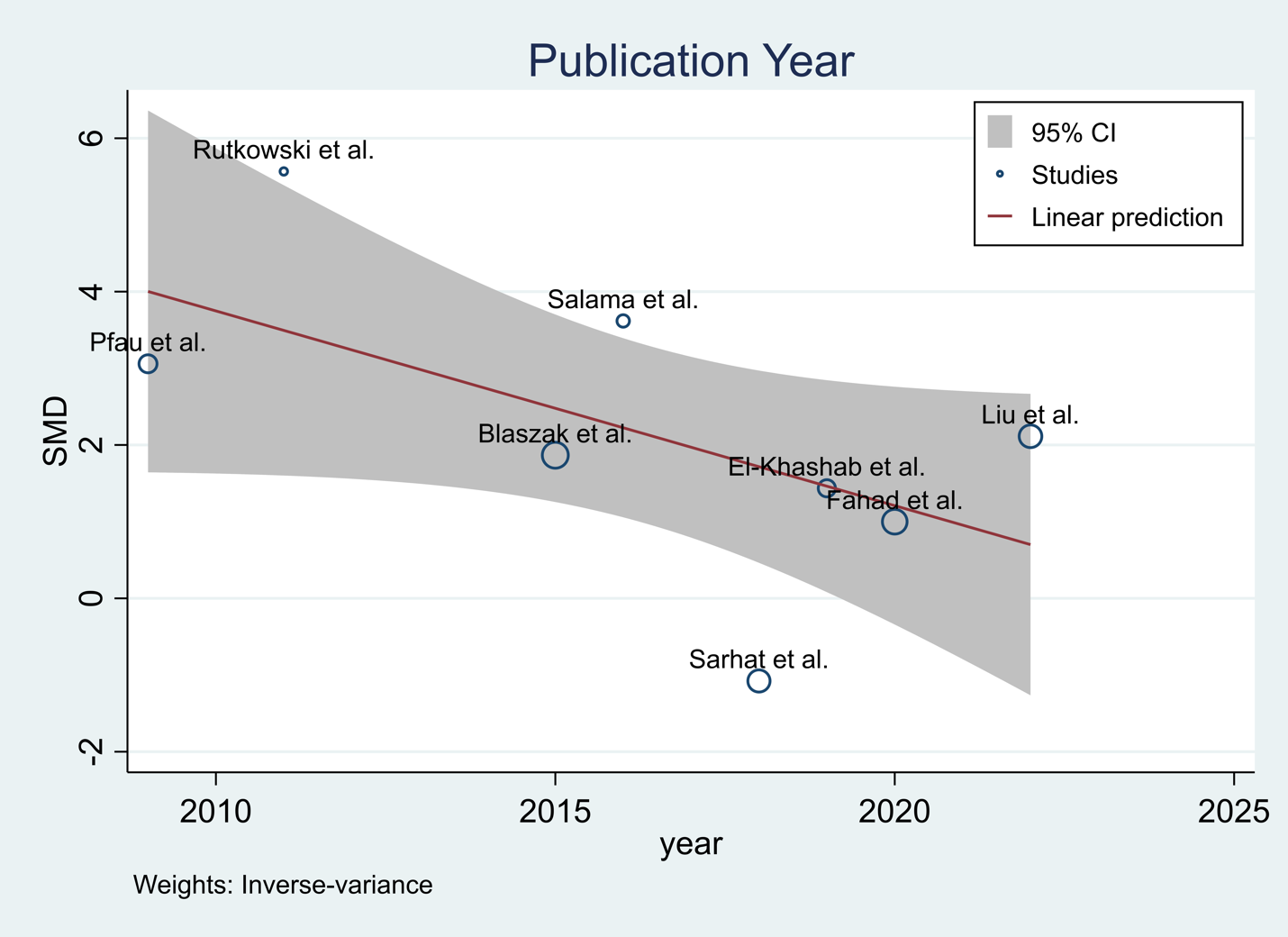


**Supplementary figure 6.** Bubble plot for meta-regression of CKD vs. controls based on publication year


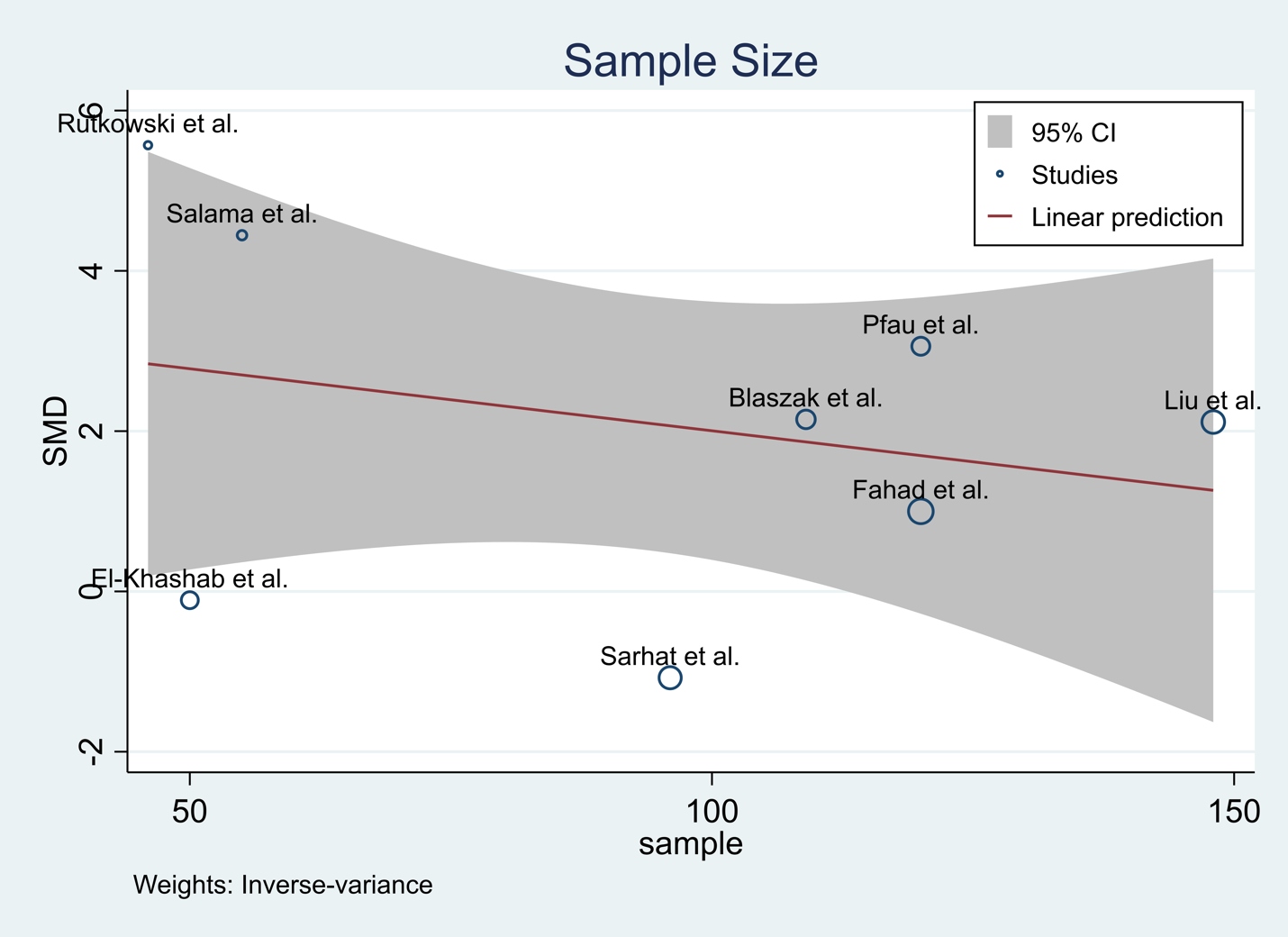


**Supplementary figure 7.** Bubble plot for meta-regression of HD vs. controls based on sample size


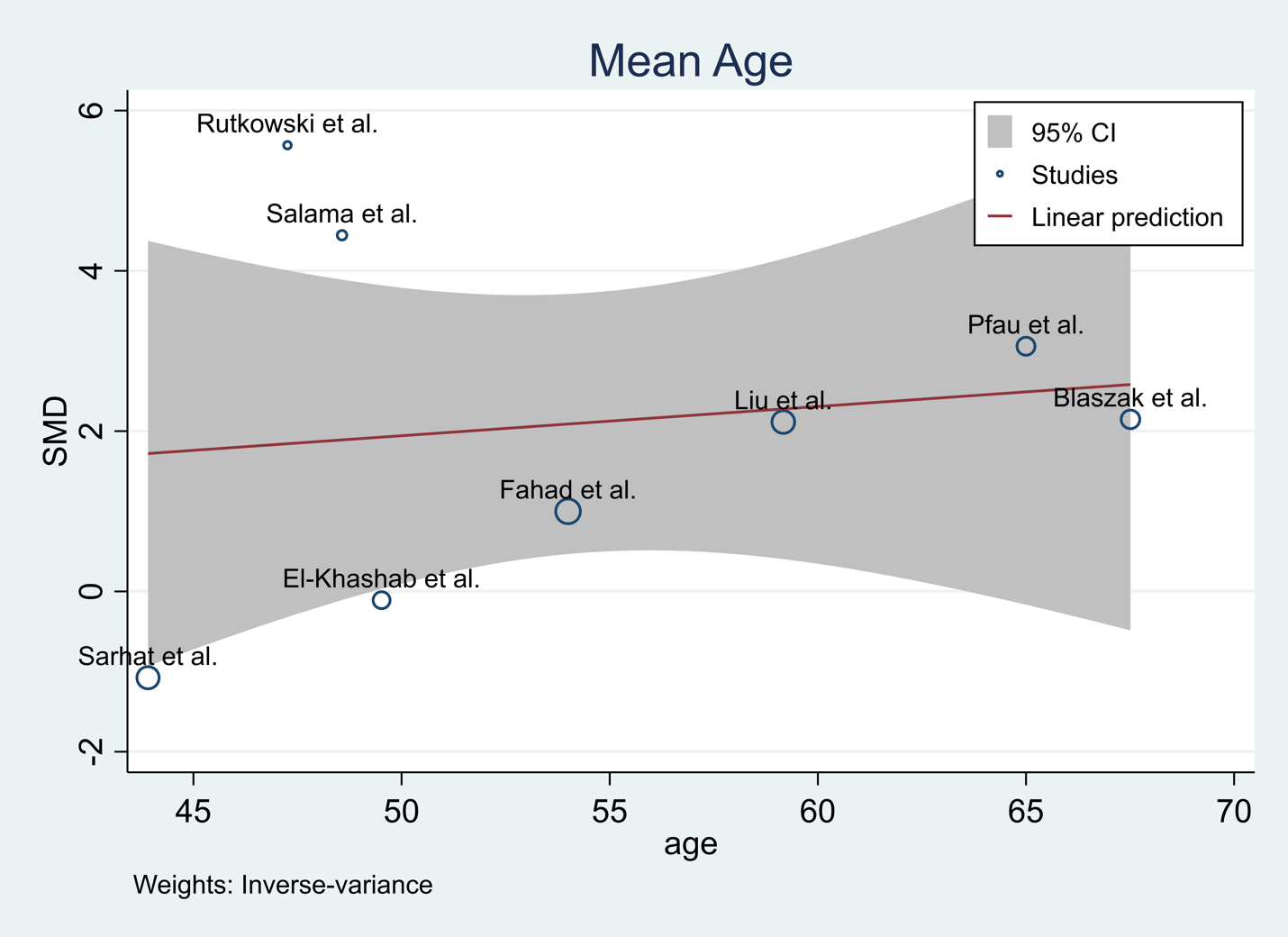


**Supplementary figure 8.** Bubble plot for meta-regression of HD vs. controls based on mean age


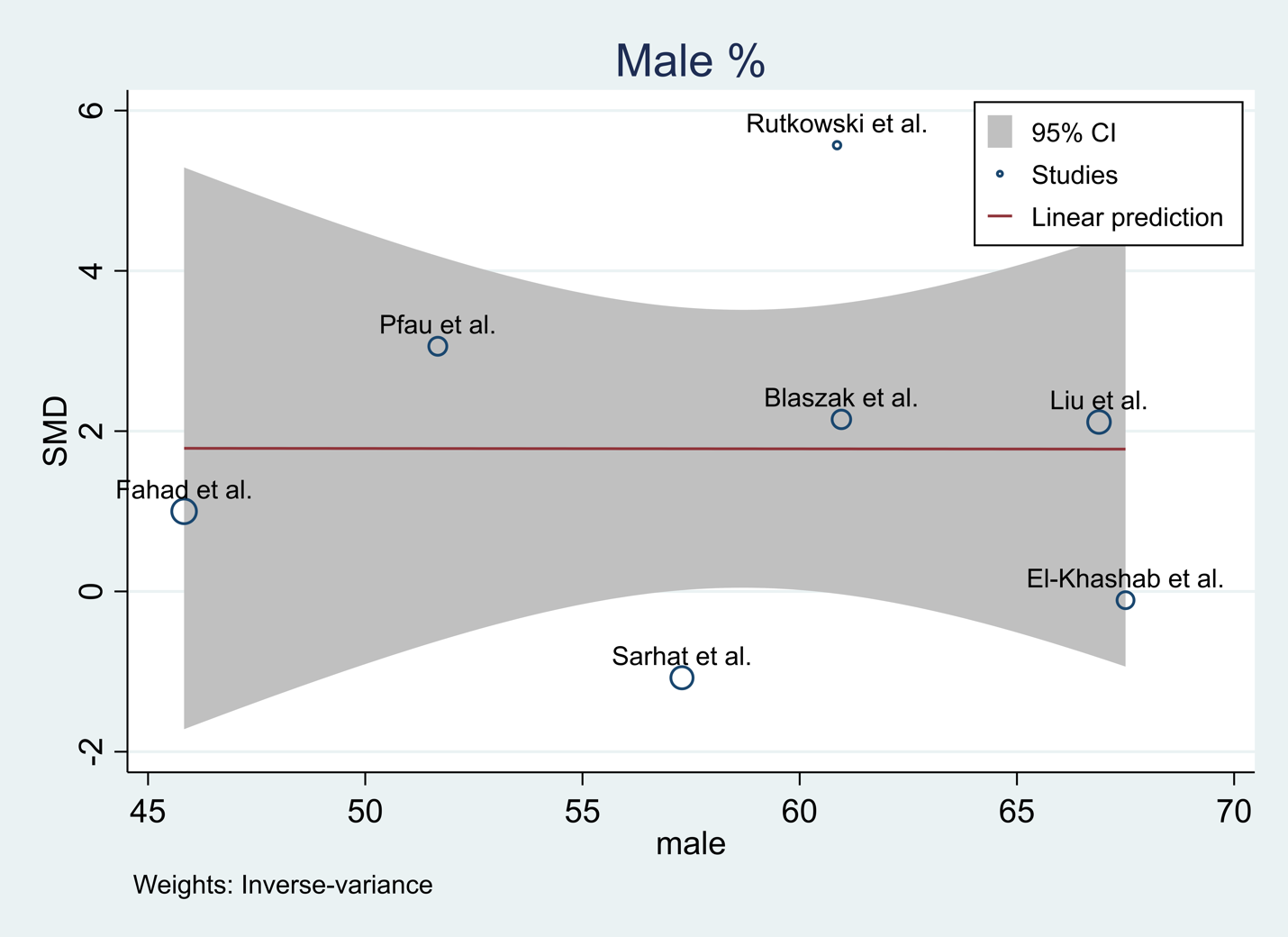


**Supplementary figure 9.** Bubble plot for meta-regression of HD vs. controls based on male percentage


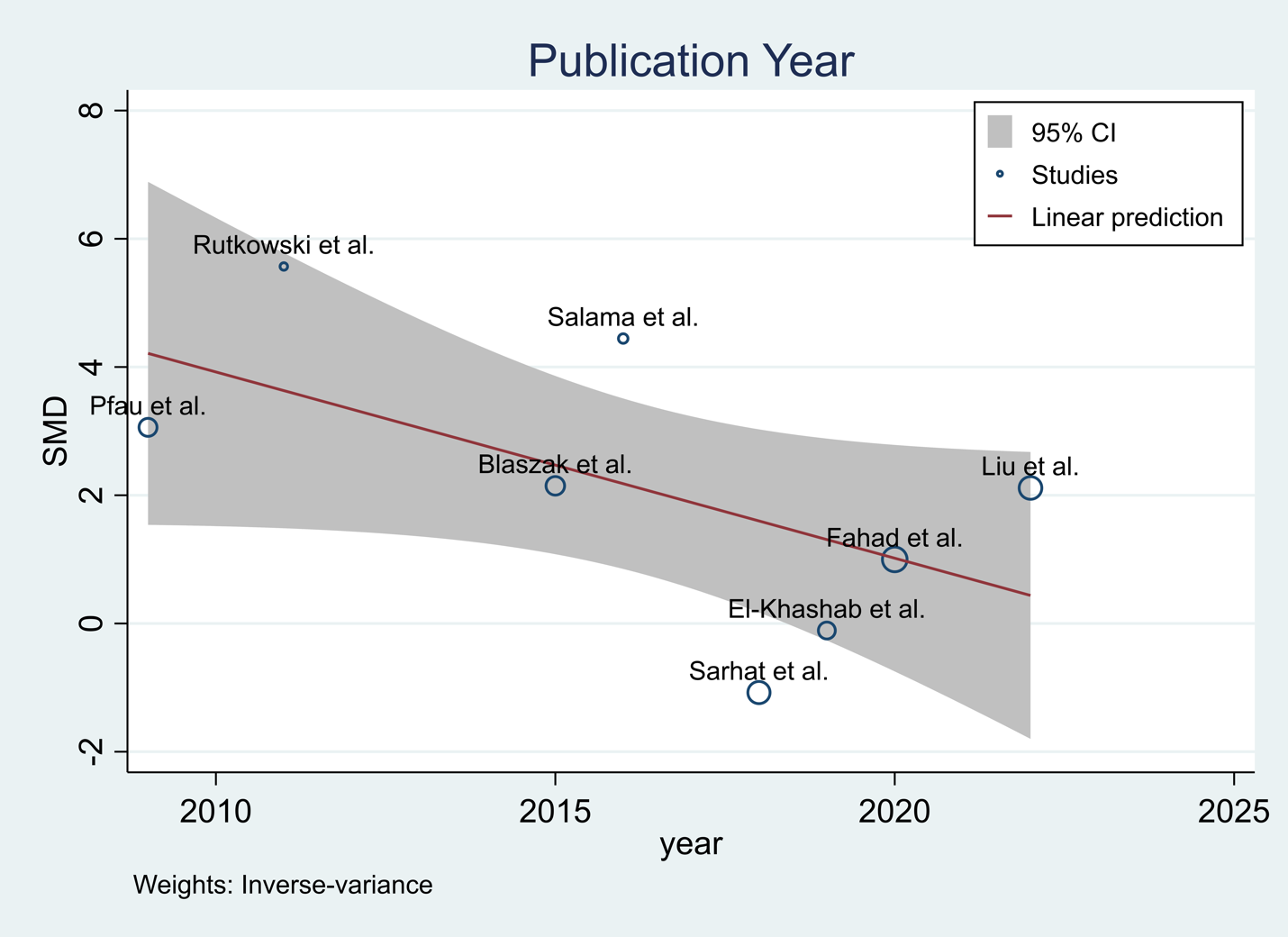


**Supplementary figure 10.** Bubble plot for meta-regression of HD vs. controls based on publication year


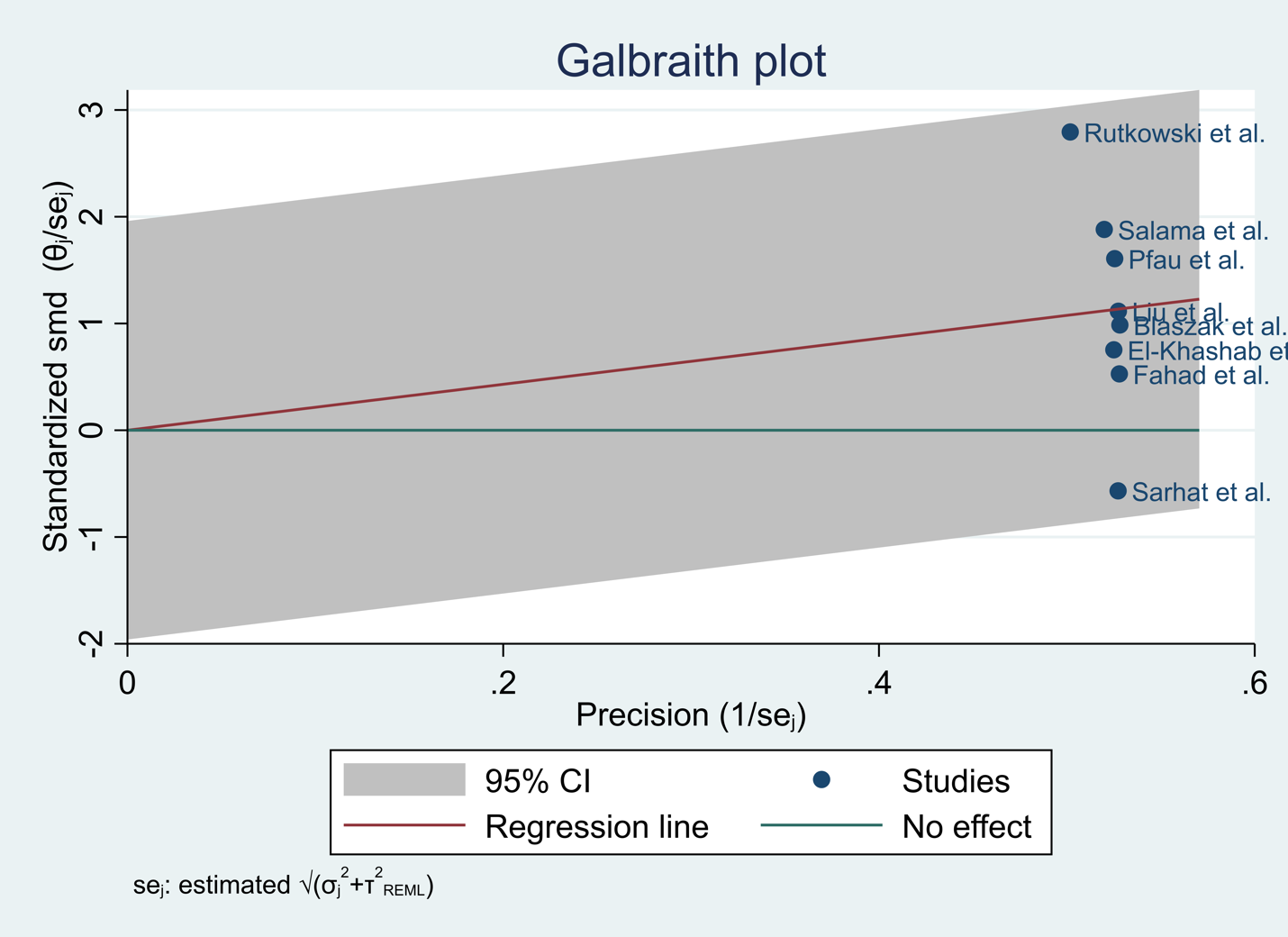


**Supplementary figure 11.** Galbraith plot for meta-analysis of CKD vs. controls


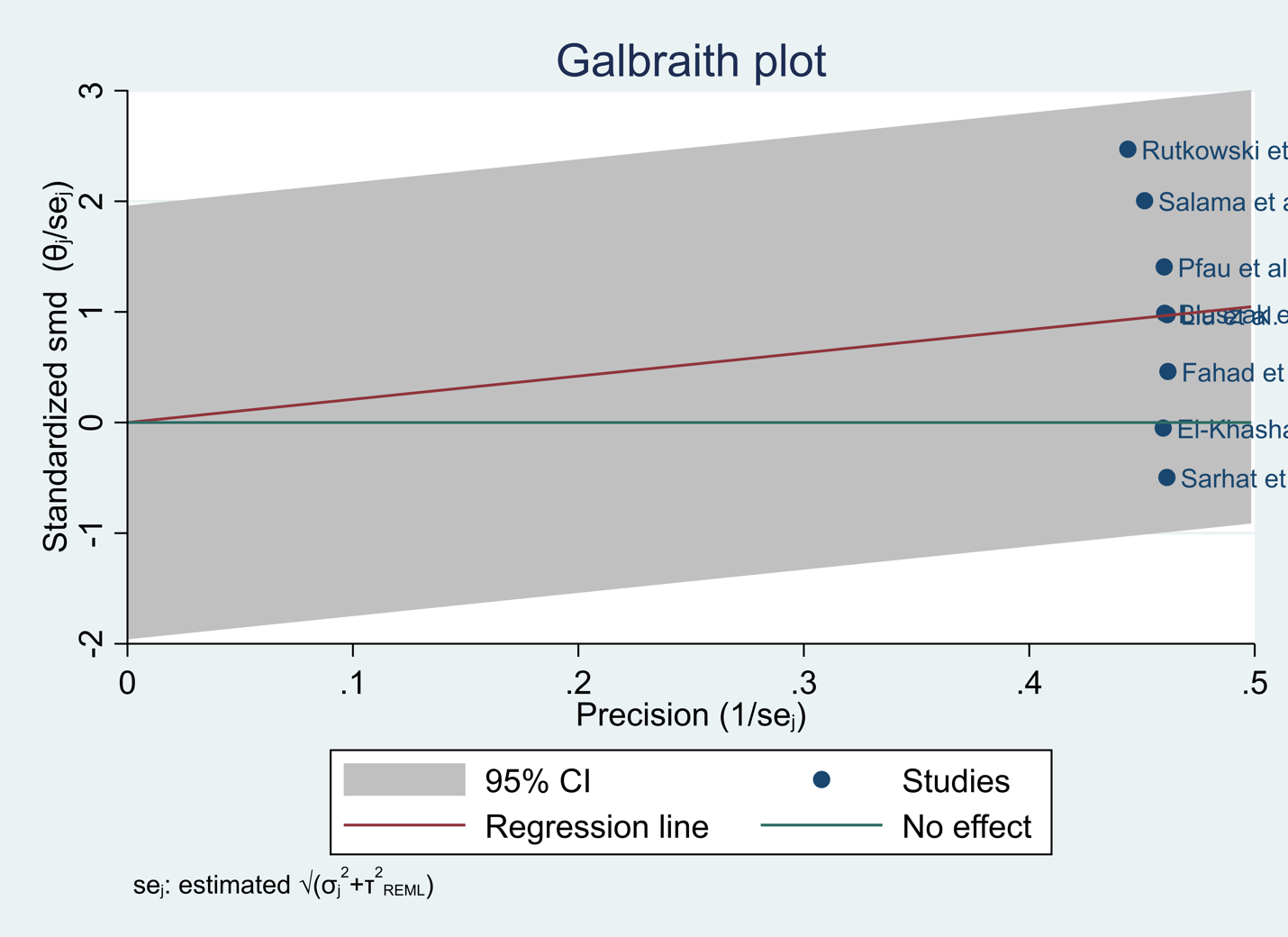


**Supplementary figure 12.** Galbraith plot for meta-analysis of HD vs. controls
